# The virus-induced protein APOBEC3G inhibits anoikis by activation of Akt kinase in pancreatic cancer cells

**DOI:** 10.1038/srep12230

**Published:** 2015-07-16

**Authors:** Jia Wu, Tian-Hui Pan, Song Xu, Li-Tao Jia, Lin-Lin Zhu, Jian-Shan Mao, Yong-Liang Zhu, Jian-Ting Cai

**Affiliations:** 1 Laboratory of Gastroenterology, Second Affiliated Hospital of Zhejiang University, School of Medicine, Hangzhou, China; 2 Cancer Institute and Education Ministry Key Laboratory of Cancer Prevention and Intervention, Zhejiang University School of Medicine, Hangzhou, China; 3 Department of Gastroenterology, Tongde Hospital of Zhejiang Province, Hangzhou, China

## Abstract

Pancreatic cancer is one of the more common cancers with a poor prognosis. Some varieties of cancer are related to virus infection. As a virus-induced protein, APOBEC3G (A3G) presents extensive anti-virus ability, but the role of A3G in pancreatic cancer was previously unknown. The expression of A3G in pancreatic cancer was examined using TaqMan real-time qPCR, immunohistochemical and immunofluorescent staining. Subsequently, the role of A3G in pancreatic cancer was evaluated *in vivo* using the tumor xenograft model. Anoikis was detected by colony formation assay and flow cytometry *in vitro*. The Akt kinase activity and target protein PTEN were examined by co-immunoprecipitation and immunoblot. The virus-induced protein A3G was significantly up-regulated in pancreatic cancer, and the up-regulation of A3G promoted xenograft tumor formation. A3G inactivated PTEN by binding to the C2 tensin-type and PDZ domains, thereby inducing anoikis resistance through Akt activation. Our results demonstrate that the up-regulation of A3G in pancreatic cancer cells induces anoikis resistance, and they provide novel insight into the mechanism by which A3G affects the malignant behavior of pancreatic cancer cells.

Pancreatic cancer is the fourth most common cause of cancer-related deaths^1^, with a 5-year survival rate of only 1–4%^2^. The poor prognosis of pancreatic cancer is associated with its early metastasis and the resistance of pancreatic cancer-initiating cells to treatment. Anoikis resistance is an early molecular event in the development of cancer and plays an important role in metastasis^3^. Anoikis is a special form of cell apoptosis induced by disengagement of the cells from the extracellular matrix and from other cells. It plays an important role in tissue homeostasis, disease development, and tumor metastasis^4^. Multiple molecular mechanisms can induce anoikis resistance, but the activation of the phosphatidylinositol-3-kinase (PI3K)/Akt pathway is the most common route of anoikis inhibition^5^,^6^,^7^.

Initiating cells residing within tumors are the origin of pancreatic cancer proliferation, metastasis, recurrence, and drug resistance. These initiating cells are also strongly resistant to anoikis. The Wnt, Notch, and Hedgehog signaling pathways regulate the stem characteristics of pancreatic cancer-initiating cells^8^. However, the canonical Wnt, Notch, and Hedgehog pathways are not involved in anoikis resistance. The molecular mechanisms behind the strong anoikis resistance of pancreatic cancer-initiating cells need to be further elucidated.

Virus infection is involved in the initiation and development of cancer. Currently, many cancers are associated with virus infection; for example, EBV is associated with gastric cancer, HBV and HCV are associated with liver and pancreatic cancer, HBV is associated with cholangiocarcinoma, HPV and human endogenous retroviruses (HERVs) are associated with colorectal cancer^9^,^10^,^11^,^12^,^13^,^14^. After infection with DNA tumor viruses such as HPV, HBV, and EBV, viral genomes are integrated into the host DNA, resulting in cell transformation. In contrast, RNA tumor viruses, also known as retroviruses, have promoters or enhancers that are meant to promote the transcription of viral proteins. These promoters and enhancers can insert into nearby oncogenes of the host DNA after reverse transcription and induce the activation and overexpression of oncogenes and can also lead to cell transformation^15^,^16^,^17^. Apolipoprotein-B mRNA-editing enzyme catalytic polypeptide (APOBEC) family proteins are critical restriction factors for limiting retroviral infection; with their cytosine deaminase activity, these proteins can cause cytosine (C) to uracil (U) hypermutations in the minus-strand of viral DNA, thus limiting the viral replicative capacity^18^,^19^,^20^. However, in addition to their anti-virus ability, APOBEC family proteins also play important roles in colon cancer metastasis^21^. The participation of APOBEC family proteins in anoikis resistance of pancreatic cancer cells was previously unknown.

Here, we found that APOBEC3G (A3G) was up-regulated in pancreatic cancer and promoted tumor formation *in vivo*. Furthermore, we found that A3G induced anoikis resistance through Akt activation, which was allowed by the inactivation of PTEN phosphatase, suggesting that A3G can enhance the malignant behavior of pancreatic cancer in addition to limiting viral replication.

## Results

### A3G is up-regulated in pancreatic cancer cells

**Figure 1:**
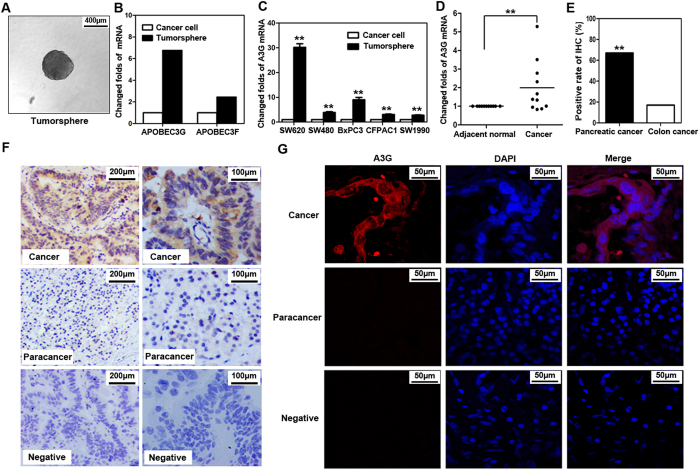
A3G is up-regulated in pancreatic cancer cells. (**A**) Tumorsphere formation. SW620 cells were cultured with serum-free cloning media at 37 °C in a 5% CO_2_ atmosphere. Tumorspheres were observed after 7–10 days (Scale bar 400μm). (**B**) cDNA expression profile microarray screening. The APOBEC3 family (*APOBEC3G* and *APOBEC3F*) were up-regulated in tumorspheres compared with attached cancer cells in cDNA expression profile microarray screening. (**C**) Real-time qPCR quantification of *A3G* in cancer cells. The expression of *A3G* was analyzed using TaqMan real-time qPCR. *A3G* mRNA was higher in tumorspheres than in attached cancer cells. All data are represented for triplicate experiments (**P < 0.01, Student’s *t-*test). (**D**) The expression of *A3G* in pancreatic cancer tissues. Total RNA was extracted from 11 matched human pancreatic cancer and *para*-cancerous tissues and subjected to TaqMan real-time qPCR. The expression of *A3G* in pancreatic cancer tissues was higher than that in *para*-cancerous tissues (**P < 0.01, Student’s *t-*test). (**E**) Expression differences of A3G in colorectal and pancreatic cancer. The expression of A3G in pancreatic cancer was higher than colon cancer in immunohistochemical staining (**P < 0.01, χ^2^ test). (**F**) Immunohistochemical staining. Slides from 54 matched human pancreatic cancer and *para*-cancerous tissues were checked by immunohistochemical staining. The expression of A3G was higher in cancer tissues (ductal) than in *para*-cancerous tissues (acinar) (Left: Scale bar 200 μm; Right: Scale bar 100 μm). (**G**) Immunofluorescent staining. Representative immunofluorescent staining showed that A3G was higher in pancreatic cancer tissues and was mainly distributed in the cytoplasm (Scale bar 50 μm).

Cancer-initiating cells have the characteristics of the malignant biological behavior of cancer. To determine the involvement of virus-induced proteins in cancer, we collected the colorectal cancer SW620-initiating cells via the serum-free tumorsphere formation assay (Fig. 1A), as described in Methods. We found that two genes of virus-induced proteins, *A3G* and *APOBEC3F* (*A3F*), were significantly enriched in SW620-initiating cells, as determined by a global cDNA expression microarray (Fig. 1B; Supplementary Table S1). Due to the higher expression of A3G relative to A3F and the stronger hyper-mutation ability of A3G^22^,^23^, our study focused on the effects of A3G. We first examined *A3G* expression using TaqMan real-time qPCR; the *A3G* mRNA levels were higher in tumorspheres than in attached cancer cells including SW480, SW620, SW1990, BxPC3, and CFPAC1 (Fig. 1C). To determine the expression of *A3G* in clinical specimens, we analyzed *A3G* expression in 11 pairs of matched human fresh pancreatic cancer tissues by real-time qPCR and found that *A3G* in pancreatic cancer tissues was significantly higher than in *para*-cancerous tissues (*P *< 0.01, Student’s *t-*test) (Fig. 1D). Although the expression of A3G is higher in tumorspheres from colon cancer, A3G expression in clinical specimens of colon cancer is much lower than that of pancreatic cancer in our study, as determined by immunohistochemical staining (*P *< 0.01, χ^2^ test) (Fig. 1E; Supplementary Fig. S1), which is similar with the data in the Human Protein Atlas (www.proteinatlas.org). Further research on A3G in pancreatic cancer is more clinically valuable. We retrospectively analyzed 54 pairs of human pancreatic cancer tissue biopsy slides by immunohistochemical staining and found that A3G expression in pancreatic cancer tissues was higher than that in matched adjacent tissues. A3G expression had no significant correlation with pancreatic cancer staging, typing or prognosis (Fig. 1F; Supplementary Table S2). Finally, we observed the expression of A3G under a confocal microscope and found that it mainly distributed in the cytoplasm (Fig. 1G; Supplementary Fig. S2). Collectively, these results show that the expression of A3G is elevated in cancer-initiating cells and pancreatic cancer cells, suggesting that A3G may participate in the initiation and development of pancreatic cancer.

### Overexpression of A3G promotes xenograft tumor formation in nude mice

**Figure 2:**
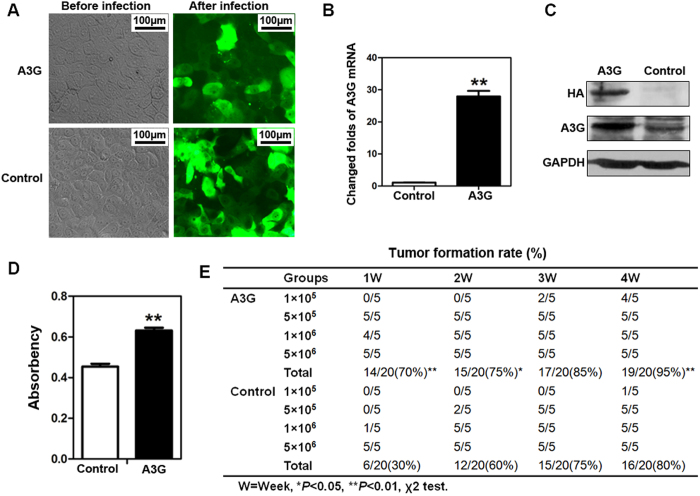
The overexpression of A3G promotes the xenograft tumor formation rate but inhibits tumor growth in nude mice. (**A**) Establishment of stable A3G expressing BxPC3 cells. BxPC3 Cells with stable A3G expression were established by recombinant lentivirus infection (Scale bar 100 μm). (**B,C**) The detection of A3G in stably expressing BxPC3 cells. Real-time qPCR and western blots revealed that *A3G* expression was significantly higher in stable A3G-expressing BxPC3 cells than in control cells (**P < 0.01, Student’s *t-*test). (**D**) Determination of cell proliferation. Cell proliferation was quantified by MTS assay. The data are represented as the means ± SEM of triplicate experiments (**P < 0.01, Student’s *t*-test). (**E**) Overexpression of A3G promotes the tumor formation rate. A3G-expressing BxPC3 cells and control cells were injected subcutaneously into nude Balb/c mice, and mice were followed for 4 weeks. The tumor formation rate was significantly higher in the A3G-expressing group than in controls (*P < 0.05, **P < 0.01, χ^2^ test).

To demonstrate the tumorigenic effect of A3G on pancreatic cancer, we established pancreatic cancer BxPC3 cells that stably express A3G (Fig. 2A). The expression of A3G was significantly elevated in stable A3G-expressing cells compared with control cells, as determined by western blot and real-time qPCR (Fig. 2B,C). (Full length blot is shown in Supplementary Fig. S3). We found that cell proliferation was obviously increased in stable A3G-expressing cells compared with control cells by MTS assay (*P *< 0.01, Student’s *t*-test) (Fig. 2D). A3G-expressing and control cells were injected into the flanks of nude mice at four increasing quantities, the results showed that 5 × 10^5^, 1 × 10^6^ and 5 × 10^6^ quantities of the A3G-expressing group could form tumors after one week, while in the control group, only 1 × 10^6^ and 5 × 10^6^ quantities of control group could form tumors. Interestingly, 1 × 10^5^ quantity of the A3G-expressing group could form more xenograft tumor than the control group after 4 weeks. A3G expression promoted rapid xenograft tumor formation in the early stage, and the total xenograft tumor-formation rate of A3G-expressing cells was significantly higher than that of control cells in a four-week experiment (*P < 0.05, **P < 0.01, χ^2^ test). (Fig. 2E; Supplementary Fig. S4). There were no significant differences in proliferation, apoptosis rate and vessel density between A3G-expressing groups and control groups in xenograft tumors (Supplementary Fig. S5). Overall, these data demonstrate that the overexpression of A3G promotes xenograft tumor formation.

### The up-regulation of A3G induces anoikis resistance

**Figure 3:**
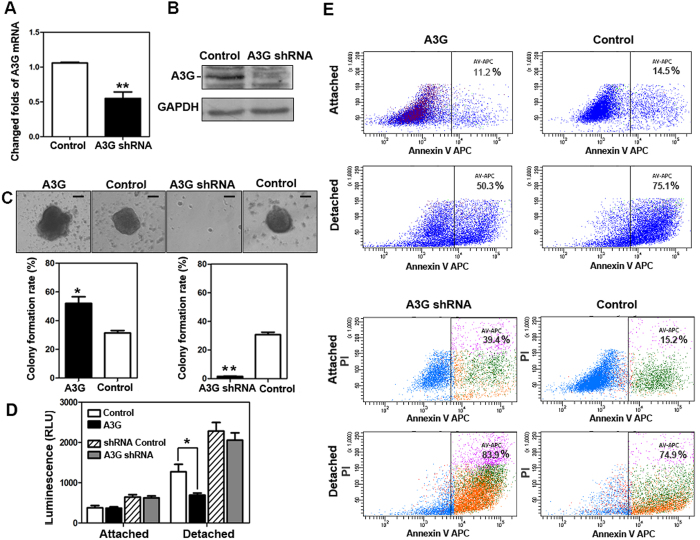
The up-regulation of A3G induces anoikis resistance. **(A,B)** The establishment of stable A3G knockdown BxPC3 cells. BxPC3 cells with stable A3G knockdown were established by recombinant lentiviral infection. Real-time qPCR and western blots revealed that A3G levels were significantly lower in stable A3G knockdown BxPC3 cells than in controls (**P < 0.01, Student’s *t*-test). (**C**) A3G expression increases the colony formation rate. Representative images from the colony formation assay for stable A3G-expressing BxPC3 cells and A3G knockdown BxPC3 cells are shown in the upper panels. The colony formation rate of A3G-overexpressing cells was significantly higher than control cells, while the colony formation rate of A3G knockdown cells was significantly lower than control cells. All data are represented as the means ± SEM of triplicate experiments (*P < 0.05, **P < 0.01, ANOVA) (Scale bar 100 μm). (**D**) Determination of Caspase 3/7 activities. Caspase 3/7 activities were inhibited in stable A3G-expressing cells after detachment (*P < 0.05, Student’s *t*-test). (**E**) A3G induces anoikis resistance. Stable A3G-expressing BxPC3 cells and A3G knockdown BxPC3 cells were stained with Annexin V/APC in attached and detached states and were subjected to flow cytometry analyses. In the detached state, stable A3G-expressing cells showed suppressed anoikis (upper 4 panels); meanwhile, stable A3G knockdown cells showed the promotion of anoikis after detachment (lower 4 panels).

To investigate the molecular mechanisms of early promotion of tumor formation by A3G, we established stable A3G knockdown BxPC3 cells by recombinant lentivirus infection and successfully validated them by western blot and real-time qPCR (Fig. 3A,B). (Full length blot is shown in Supplementary Fig. S6). In the colony formation assay, cancer-initiating cells resist anoikis in a serum-free suspension culture condition^24^. The stable A3G-expressing cells, A3G knockdown cells and control cells were cultured in low-adhesion plates with serum-free media. The colony formation rate of stable A3G-expressing cells was significantly higher than control cells, while the colony formation rate of A3G knockdown cells was significantly lower than control cells. (*P *< 0.05, **P < 0.01, ANOVA) (Fig. 3C). Caspase 3/7 activities were inhibited in stable A3G-expressing cells after detachment compared with control cells (*P *< 0.05, Student’s *t*-test), but had no statistically significant difference in the A3G knockdown cells (Fig. 3D). To further validate the impact of A3G on anoikis, we detected the percentage of Annexin V-positive cells in the A3G-expressing cells and the A3G knockdown cells in attachment and detachment conditions by flow cytometry (FCM) analysis. The results showed that the percentage of Annexin-positive stable A3G-expressing cells after detachment was lower than that during attachment, indicating that A3G could inhibit early-stage apoptosis; meanwhile, A3G knockdown promoted apoptosis after detachment (Fig. 3E). Collectively, these results indicate that the up-regulation of A3G promote anoikis resistance.

### A3G promotes anoikis resistance through the PTEN-mediated activation of Akt kinase

**Figure 4:**
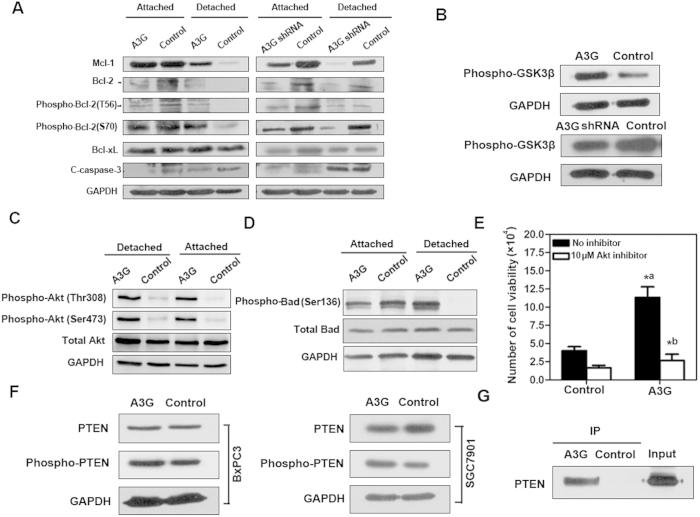
A3G promotes anoikis resistance through PTEN-mediated activation of the Akt pathway. (**A**) Apoptosis-related proteins are involved in anoikis resistance. Western blot analysis of apoptosis-related proteins in stable A3G-expressing cells, A3G knockdown cells and control cells. Compared with the control cells, the anti-apoptotic proteins Bcl-2, Mcl-1 and phospho-Bcl-2 were up-regulated and cleaved caspase-3 was attenuated in stable A3G-expressing cells after detachment. In contrast, Bcl-2, Mcl-1 and phospho-Bcl-2 were down-regulated in stable A3G knockdown cells. GAPDH was used as a loading control. (**B**) Akt kinase is activated by A3G. Akt kinase activity was up-regulated in stable A3G-expressing BxPC3 cells. GAPDH was used as a loading control. (**C**) Detection of phospho-Akt. A3G-expressing cells and control cells were collected and seeded into poly-HEMA-coated 6-well plates in attachment and detachment conditions. The cells were incubated at 37 °C for 24 h. Western blot analysis of Akt in A3G-expressing cells and control cells. Phospho-Akt (Thr308) and phospho-Akt (Ser473) were up-regulated in A3G-expressing cells compared with control cells, both in attachment and detachment. GAPDH was used as a loading control. (**D**) Detection of phospho-Bad. Western blot analysis of Bad in stable A3G-expressing BxPC3 cells and control cells. Compared with control cells, phospho-Bad (Ser136) was up-regulated in stable A3G-expressing cells after detachment. GAPDH was used as a loading control. (**E**) Inhibition of Akt decreases anoikis resistance in A3G overexpressing cells. Cell viability of stable A3G-expressing BxPC3 cells after detachment was significantly higher than control (*^a^*P *< 0.05; Student’s *t*-test); cell viability of stable A3G-expressing BxPC3 cells in detached state was remarkably decreased by Akt inhibition (*^b^*P *< 0.05; Student’s *t*-test). All data are represented as the means ± SEM for triplicate experiments. (**F**) Detection of PTEN. Western blot analysis demonstrated that phospho-PTEN was up-regulated in stable A3G-expressing BxPC3 cells and SGC7901 cells transiently transfected with *A3G*-*HA* plasmid. (**G**) PTEN is a target of A3G-induced Akt activation. Stable A3G-expressing BxPC3 cells were lysed, and A3G was co-immunoprecipitated with HA-tag agarose-conjugated antibodies. Western blot detection of PTEN showed that A3G could interact with PTEN. All data are repeated for three times in the same condition.

To clarify the molecular mechanism of anoikis resistance induced by A3G overexpression, we detected apoptosis-related proteins and Akt activity in stable A3G-expressing cells, A3G knockdown cells and control cells by western blots. We found that the anti-apoptotic proteins Bcl-2, Mcl-1 and phospho-Bcl-2 were up-regulated, and the apoptotic protein cleaved caspase-3 was down-regulated in stable A3G-expressing cells after detachment compared with control cells, while protein Bcl-xL was unchanged. In contrast, Bcl-2, Mcl-1 and phospho-Bcl-2 were down-regulated in stable A3G knockdown cells (Fig. 4A). In addition, the Bcl-2 family pro-apoptotic protein Bid was elevated in A3G-expressing cells, while Bmf was unchanged (Supplementary Fig. S7). A3G expression level in attached and detached cells was similar (Supplementary Fig. S8). On the other hand, Akt kinase activity was up-regulated in A3G-expressing cells and down-regulated in A3G knockdown cells (Fig. 4B). The expression of phospho-Akt (Thr308) and phospho-Akt (Ser473) were up-regulated in A3G-expressing cells compared with control cells, both in attachment and detachment (Fig. 4C). These results indicated that A3G could activate the Akt pathway. Activited Akt increased the phosphorylation at Ser-136 of Bad in stable A3G-expressing cells after detachment (Fig. 4D). Further, inactivation of Akt by a chemical inhibitor significantly decreased the A3G-expressing cell viability, indicating that anoikis resistance in A3G overexpressing cells mainly depends on the activation of Akt (Fig. 4E). Akt activation can induce anoikis resistance^25^. Phosphorylation of PTEN, a key inhibitor of the Akt pathway, reduces its activity. Western blot analysis showed that phospho-PTEN protein was increased in stable A3G-expressing BxPC3 cells and SGC7901 cells that were transiently transfected with A3G, indicating that the up-regulation of A3G can inhibit the activity of PTEN (Fig. 4F). To further clarify the effect of A3G on PTEN, we detected the interaction of A3G and PTEN by co-immunoprecipitation (Fig. 4G). (Full length blot is shown in Supplementary Fig. S9, 10, 11).These data suggest that the up-regulation of A3G activates the Akt pathway through the inactivation of PTEN, leading to anoikis resistance in pancreatic cancer.

### The interaction of A3G and PTEN depends on the binding domain

**Figure 5:**
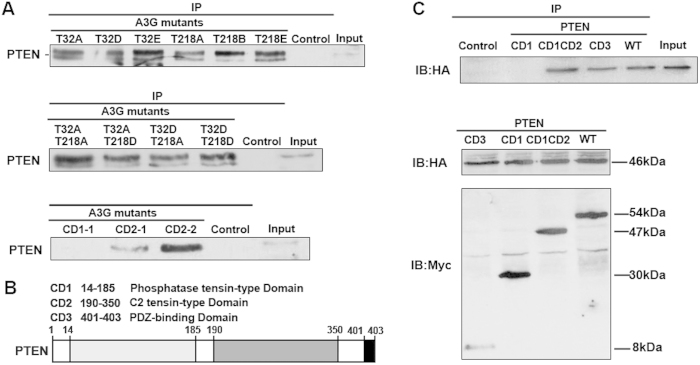
The interaction of A3G and PTEN depends on the binding domain. (**A**) The binding capacity of A3G and PTEN is related to a zinc-coordinating domain of A3G but not to its phosphorylation state. PTEN was co-immunoprecipitated from 293T cells transfected with different *A3G* mutants (T32A, T32D, T32E, T218A, T218D, T218E, T32A/T218A, T32A/T218D, T32D/T218A and T32DT218D) and A3G CD mutant plasmids (CD2-1 and CD2–2). PTEN was not co-immunoprecipitated from 293T cells transfected with the A3G CD mutant plasmid CD1-1. All data are repeated for three times in the same condition. (**B**) Binding domain of PTEN. Shown is an illustration of the PTEN binding domain. (**C**) Binding capacity is related to the domain of PTEN. The top image shows that A3G HA-tagged protein was co-immunoprecipitated from 293T cells co-transfected with *A3G* HA-tagged plasmid and *PTEN* Myc-tagged CD mutant plasmids (CD1CD2, CD3) but not with PTEN CD1 mutant plasmid. The bottom image shows the input of 293T cells co-transfected with *A3G* HA-tagged plasmid and *PTEN* Myc-tagged CD mutant plasmids. All data are repeated for three times in the same condition.

The above results demonstrate that A3G interacted with the PTEN protein, but the binding sites were unknown. Using a bioinformatics prediction of phosphorylation sites, we constructed A3G phosphorylation site mutants using site-directed mutagenesis. We found that all A3G phosphorylation site mutants (T32A, T32D, T32E, T218A, T218D, T218E, T32A/T218A, T32A/T218D, T32D/T218A, and T32D/T218D) could interact with PTEN. The A3G domain mutants CD2-1 and CD2-2 also interacted with PTEN, but the A3G domain mutant CD1-1 did not (Fig. 5A). We also established PTEN mutants (Fig. 5B) and found that PTEN domain mutants CD1CD2 and CD3 but not domain mutant CD1 could interact with A3G (Fig. 5C). These results indicate that the interaction of A3G and PTEN requires the presence of the C2 tensin-type domain and PDZ domain of PTEN and the CD2 domain of A3G.

## Discussion

Our study found that the expression of the virus-induced protein A3G in pancreatic cancer was significantly greater than that in normal pancreatic tissue but had no significant correlation with pancreatic cancer staging, typing or prognosis. A3G promoted cell proliferation of pancreatic cancer cells *in vitro* and the formation of pancreatic tumors in the early stage *in vivo*. A3G activated Akt through inactivation of PTEN, inducing anoikis resistance *in vitro*. These results indicate that A3G, in addition to its anti-virus activity, is involved in the regulation of the malignancy of pancreatic cancer.

There were insignificant differences in proliferation, apoptosis rate and vessel density between A3G-expressing groups and control groups *in vivo* experiments. The possible reason A3G-expressing group could form tumors faster is that A3G improved the sensitivity of pancreatic cells to form tumors. A3G inhibited anoikis by activation of Akt. This phenomenon may be due to the complicated cluster of factors affecting *in vivo* experiments. In our manuscript, overexpression of A3G promotes xenograft tumor formation in the early stage, there may be other factors affecting the tumor proliferation in the latter stage. So there are some differences between *in vivo* and *in vitro* experiments.

A3G, a member of the APOBEC family, can induce hypermutations in the minus-strand of viral DNA to limit viral replicative capacity^18^,^19^. The human *A3G* gene is located on the long arm of chromosome 22 (22q13.1-q13.2) and is composed of eight exons and seven introns. The encoded A3G protein of 384 amino acids has a molecular weight of 46,405 Da. A3G has two homologous zinc-coordinating domains (CD1 and CD2), which play important roles in antiviral activity such as blocking the replication of HIV and HBV^26^,^27^,^28^,^29^. The antiviral ability and role in DNA repair of A3G are well known, but the effects of A3G in solid tumors are rarely reported^18^,^19^,^30^,^31^. We found that A3G had no hypermutation activity toward eukaryotic DNA by the detection of the hypermutation of *K-Ras* DNA in eukaryotic cells overexpressing A3G (data not shown), indicating that the role of A3G in pancreatic cancer malignancy is via a novel mechanism independent of deaminase activity.

Anoikis, a special form of programmed cell death, plays an important role in tissue homeostasis, disease development, and cancer metastasis^32^. Anoikis is a self-defense mechanism for organisms to eliminate detached cells before they acquire anchorage-independent survival, which is a specific marker of cancer cells. Anoikis resistance may lead to the growth of adherent cells in a detached suspension state and ectopic proliferation in abnormal locations^9^. Cancer cells acquire anoikis resistance through several mechanisms, involving growth factors, integrins, epithelial- mesenchymal transition (EMT) activation, etc^33^. Anoikis resistance can cause the expansion and invasion of cancer cells to the surrounding tissues, eventually leading to metastasis^6^. Most pancreatic cancers are diagnosed at a metastatic stage; hence, anoikis resistance may be an important early characteristic of pancreatic cancer. Ding *et al.*^21^ found that A3G can promote liver metastasis through the inhibition of miR-29-mediated suppression of MMP2 in an orthotopic mouse model of colorectal cancer. Our results show that A3G can induce anoikis resistance in pancreatic cancer, which may be a novel mechanism allowing pancreatic cancer metastasis.

Anoikis employs both intrinsic and extrinsic apoptotic pathways. The intrinsic pathway, also known as the mitochondrial pathway, is initiated by damage to the mitochondrial membrane to promote the release of pro-apoptotic factors such as cytochrome *c* into the cytoplasm, resulting in the activation of Caspase-3 and Caspase-7. The extrinsic pathway is mediated by death receptors such as Apo1/Fas or TRAIL, inducing the formation of the death-inducing signaling complex (DISC) to further activate Caspase-8^34^.

Bcl-2 family proteins include pro-apoptotic and anti-apoptotic members; the balance between these two members affects overall cell survival and death^35^. Bcl-2 (B cell lymphoma-2) family proteins, including anti-apoptotic proteins such as Bcl-2, Bcl-xL and Mcl-1 and pro-apoptotic proteins such as Bid, Bad, Noxa, Puma, Bim, Bax, Bak, and Bok, play an important role in the mitochondrial pathway^36^,^37^,^38^,^39^,^40^. Woods *et al.* revealed that the ubiquitination and degradation of Mcl-1 could activate Bax, leading to anoikis resistance^41^. In our data, apoptosis-related proteins are involved in anoikis resistance.

Akt, also known as protein kinase B (PKB), is a serine/threonine kinase that can induce the phosphorylation of multiple transcription factors, inhibit the expression of apoptotic genes, and enhance the expression of anti-apoptotic genes, thus promoting cell survival^42^. Phosphatidylinositol 3-kinase (PI3K) activation can produce phosphotidylinositol-4,5-bisphosphate (PIP2) and phosphatidylinositol-3,4,5- trisphosphate (PIP3). PIP3 functions to activate downstream signaling via Akt, which plays a key role in cell growth and survival. Akt promotes cell survival through multiple mechanisms, one of which is preventing the release of cytochrome *c* by phosphorylation of the pro-apoptotic protein Bad, thereby inhibiting apoptosis^43^. Akt can induce the degradation of the NF-κB inhibitor IκB through the phosphorylation of IκB kinase (IKK), thereby causing the translocation of NF-κB from the cytoplasm to the nucleus and resulting in the activation of target genes that promote cell survival^44^, but the results of a luciferase activity assay demonstrate that NF-κB is not activated by A3G (Supplementary Fig. S12). In our study, overexpression of A3G activates the Akt pathway and up-regulates the anti-apoptotic proteins. In fact, Akt activation does not directly induce the up-regulation of anti-apoptotic proteins Bcl-2 and Mcl-1. Bad is a target of Akt kinase. Akt activation increases the phosphorylation of Bad Ser136 which participates in anoikis resistance. However, anoikis resistance can be inhibited by inactivation of Akt, indicating that Akt pathway plays more important role in anoikis resistance of A3G overexpressing pancreatic cancer cells.

PTEN, an important inhibitor in the Akt pathway, is a polypeptide consisting of 403 amino acids, composed of the N-terminal phosphatase tensin-type domain, the C2 tensin-type domain and the C-terminal PDZ-binding domain that is involved in the maintenance of protein stability^45^,^46^. Our study shows that the interaction of A3G with PTEN requires the presence of the C2 tensin-type and PDZ domains of PTEN and the CD2 domain of A3G. Consistent with the mechanism for the protein TrkB, which induces anoikis resistance by activating Akt^5^, A3G inhibits anoikis by Akt activation in pancreatic cancer. The sustained activation of Akt can prevent apoptosis mediated by PTEN through the phosphorylation of downstream target proteins. PTEN inhibits the activity of Akt through dephosphorylation of PIP3 into PIP2, and PTEN deletion may be an important mechanism of anoikis resistance^47^,^48^,^49^.

In conclusion, we found a novel mechanism by which the virus-induced protein A3G activates the Akt pathway through the inactivation of PTEN, which leads to anoikis resistance in pancreatic cancer cells, suggesting that A3G enhances the malignant behavior of pancreatic cancer cells in addition to limiting viral replication.

## Methods

### Cell lines

The pancreatic cancer BxPC3, CFPAC1, and SW1990 cells; colon cancer SW480 and SW620 cells; gastric cancer SGC7901 cells; and human embryonic kidney 293T cells were purchased from the cell bank of the Shanghai Branch of the Chinese Academy of Sciences. BxPC3 and SGC7901 cells were cultured in RPMI-1640 media (Gibco, Gaithersburg, MD, USA); CFPAC1 cells were cultured in IMDM media (Gibco); SW1990, SW620 and SW480 cells were cultured in L15 media (Gibco); and 293T cells were cultured in DMEM media (Gibco) with high glucose. Cell lines were cultured in media containing 100 U/ml penicillin, 100 μg/ml streptomycin, and 15% fetal bovine serum and maintained at 37 °C in a 5% CO_2_ atmosphere.

### Human samples and ethics

The 54 matched human pancreatic cancer, 23 matched human colon cancer pathological slides and 11 matched fresh pancreatic cancer and *para*-cancerous tissues were collected in the Second Affiliated Hospital of Zhejiang University School of Medicine between 2009 and 2011. This research was approved by the Institutional Review Board of the Second Affiliated Hospital of Zhejiang University School of Medicine. Informed consent was obtained from all patients in our research. All experiments were performed in accordance with relevant guidelines and regulations of china.

### Tumorsphere formation assay

Cells were seeded at 10000 cells per well on poly-HEMA-coated 6-well plates (Corning, New York, NY) in serum-free cloning media (including 2% B27, 20 μg/L EGF, 20 μg/L bFGF, 4 mg/L insulin, 0.4% BSA and 200 IU/mL penicillin-streptomycin) and cultured at 37 °C in a 5% CO_2_ atmosphere for 7–10 days. Tumorspheres were collected for further use.

### Real-time qPCR

Total RNA was extracted from cells using the RNeasy Kit (Qiagen, Hilden, Germany) or from the 11 matched human pancreatic cancer and *para*-cancerous tissues using TRIzol reagent (Invitrogen, Carlsbad, CA, USA). The RNA was reverse-transcribed using a PrimeScript RT reagent kit (Takara, Tokyo, Japan) according to the manufacturer’s instructions and then subjected to PCR amplification using Premix Ex Taq (Takara) (95 °C for 30 s, followed by 40 cycles of 95 °C for 5 s and 60 °C for 34 s) on a TaqMan real-time qPCR system (Applied Biosystems, CA, USA). The relative quantities of *A3G* RNA were normalized to relative quantities of *GAPDH* and analyzed using the △△Ct method with 95% confidence. The primers and probe for *A3G* (top: 5′-CAACCAGGCTCCACATAAAC-3′, bottom: 5′-GGTGAAGCAGGTAACCCT GT-3′, probe: 5′Fam- CTCTGCATGGCGGCCT TCAA-3′Tamra) and *GAPDH* (top: 5′-TGGTATCGTGGAAGGACTCA-3′, bottom: 5′-CCAGTAGAGGCAGGGATGAT-3′, probe: 5′Fam-ACGCCACAGTTTCCCGGAGG-3′ Tamra) were chemically synthesized (Sangon, Shanghai, China) for TaqMan real-time qPCR.

### Immunohistochemical and immunofluorescent staining

Pathological slides from 54 matched human pancreatic cancer and *para*-cancerous tissues were used for immunohistochemical and immunofluorescent staining. Pathological slides from 23 matched human colon cancer and para-cancerous tissues were used for immunohistochemical staining. The slides were incubated at 60 °C overnight and retrieved in antigen retrieval solution (PH 6.0) at 95 °C for 15 min after deparaffination. The slides were incubated with blocking buffer (10% goat serum in TBST) for 1 h, rinsed in TBST 3 times, and incubated with anti-A3G antibody (Epitomics, CA, USA) at a 1:50 dilution at 4 °C overnight. HRP-conjugated goat anti-rabbit secondary antibody or fluorescein goat anti-rabbit IgG DyLight 633 secondary antibody (Epitomics) was added at a 1:200 dilution for 1 h. For immunohistochemical analysis, slides were stained with DAB and observed under a microscope; for immunofluorescent staining, nuclei were stained with DAPI (Invitrogen, Carlsbad, CA). The expression and localization of A3G proteins were observed under a confocal microscope system (Carl Zeiss, Jena, Germany).

### Lentivirus infection

To establish a pancreatic cancer cell line that stably expresses *A3G* or *A3G* shRNA, BxPC3 cells were infected with the supernatant of cells that had been transfected with the cDNA of *A3G* genes in pLenO-DLE-Puro vectors or with *A3G* shRNA lentiviral particles (Santa Cruz Biotechnology, Santa Cruz, CA), respectively. BxPC3 cells were infected with the relevant vector as control cells. The stable clones expressing *A3G* with HA-tag or *A3G* shRNA were selected using puromycin (1.6 μg/mL). The efficiency of overexpression or knockdown was analyzed by western blot with the A3G antibody (1:500, Epitomics, CA, USA).

### Proliferation assay

Cells were washed with serum-free RPMI-1640 medium and incubated overnight under a serum-free condition. The synchronized cells were seeded into a 96-well plate (0.2 × 10^4^/well) and incubated at 37 °C under an atmosphere of 5% CO_2_ for 24 h. 3-(4,5-dimethylthiazol-2-yl)-5-(3-carboxymeth-oxyphenyl)-2-(4-sulfophenyl)-2H-tetrazolium, inner salt (MTS) reagent (Promega, Madison, WI) were added into the wells (20 μl/well). The absorbance (A) of each well was measured at 2 h by a plate reader at a wavelength of 490 nm. The proliferation rate was calculated as ΔA =A_2h_-A_0h_.

### Animal tumorigenicity experiments

All methods were carried out in accordance with the Guidelines for the Care and Use of Laboratory Animals of the Council of Science and Technology of China. All experimental protocols were approved by the Animal Care and Use Committee of Second Affiliated Hospital of Zhejiang University School of Medicine. Forty male four-week old BALB/c athymic nude mice were purchased from the Shanghai Laboratory Animal Center of the Chinese Academy of Sciences. BxPC3 cells stably expressing A3G and control cells were harvested and resuspended in 200 μl serum-free media. Different numbers of cells (1 × 10^5^, 5 × 10^5^, 1 × 10^6^ or 5 × 10^6^) were subcutaneously injected into the flank of nude mice. The growth of the xenografted tumors was observed for four weeks.

### Colony formation assay

Stable A3G-expressing cells, stable A3G knockdown cells and control cells were collected and seeded into poly-HEMA-coated 24-well plates with serum-free cloning media (including 2% B27, 20 μg/L EGF, 20 μg/L bFGF, 4 mg/L insulin, 0.4% BSA and 200 IU/mL penicillin-streptomycin). All cells were cultured in a gradient of 10 cells per well, 50 cells per well, 200 cells per well and 500 cells per well, followed by incubation at 37 °C in a 5% CO_2_ atmosphere for 7 days. The colony formation efficiency was calculated with the following formula: Efficiency = Clones / Cell numbers × 100%.

### Caspase 3/7 activity assay

Stable A3G-expressing cells, A3G knockdown cells and control cells were collected and seeded into poly-HEMA-coated 24-well plates in attachment and detachment conditions. The cells were incubated at 37 °C for 24 h. Caspase 3/7 activity assay was performed with Caspase-Glo 3/7 Assay (Promega) according to the manufacturer’s instructions.

### Flow Cytometry

Stable A3G-expressing cells, stable A3G knockdown cells and control cells were digested with 0.25% trypsin, collected before or after detachment by centrifugation at 1000 rpm for 5 min, washed twice with cold PBS and resuspended in 1× binding buffer (0.1 M HEPES, 1.4 M NaCl, 25 mM CaCl_2_). The solution (including 1 × 10^5^ cells) was transferred to a 1.5-ml Eppendorf tube, and 5 μl of APC Annexin V (BD Biosciences, California, USA) and 5 μl of PI (for two-color analysis only) were added, followed by incubation at 25 °C in the dark for 15 min. To each tube, 400 μl of 1× binding buffer was added, and the cells were subjected to flow cytometry (BD, NJ, USA) analysis within 1 h.

### Western blot and Co-Immunoprecipitation

The cells were collected and combined with 500 μl of radio-immunoprecipitation assay (RIPA) lysis buffer (1% NP-40, 0.25% sodium deoxycholate, 5 mM DL-dithiothreitol (DTT), 1× protease inhibitor cocktail (Merck, NJ, USA)) for 15 min. The supernatant was collected by centrifugation at 13,000 rpm for 10 min and subjected to 12% sodium dodecyl sulfate polyacrylamide gel electrophoresis (SDS-PAGE). For co-immunoprecipitation, the supernatants were mixed with HA-tag or Myc-tag agarose-conjugated antibodies. The immunoprecipitates were washed five times with RIPA lysis buffer and then separated by SDS-PAGE. After the proteins were transferred to nitrocellulose membranes (Whatman, Dassel, Germany), the membranes were blocked with 5% non-fat milk for 1 h and incubated with primary antibodies at room temperature for 1 h or overnight at 4 °C. The primary antibodies used in the study were as follows: Mcl-1, Bcl-2, phospho-Bcl-2 (T56), phospho-Bcl-2 (S70), Bcl-xL, Caspase-3, phospho-Akt (Thr308), phospho-Akt (Ser473), total Akt, Bad, phospho-Bad (Ser136), PTEN, phospho-PTEN (all 1:1000; Cell Signaling Technology, Danvers, MA, USA); GAPDH (1:5000, KangChen Biotech, Shanghai, China), and HA-tag and Myc-tag antibodies (1:1000, MBL, Nagoya, Japan). The membranes were washed and incubated with secondary antibodies (horseradish peroxidase-labeled goat anti-rabbit antibody) (1:2000; Cell Signaling Technology) at room temperature for 1 h. The reactive bands were detected using enhanced chemiluminescence (ECL) (Cell Signaling Technology).

### Viable cell counts

Stable A3G-expressing cells and control cells were collected and seeded into poly-HEMA-coated 24-well plates. Each group was cultured with and without 10 μM Akt inhibitor IV (Merck, NJ, USA), respectively. All cells were cultured in the concentration of 2 × 10^5^ cells per well, followed by incubation at 37 °C in a 5% CO_2_ atmosphere for 24 h. The cells were counted and evaluated the survival with Trypan blue dying.

### Akt kinase assay

Stable A3G-expressing cells, stable A3G knockdown cells and control cells were collected, followed by the addition of agarose-conjugated antibodies. Twenty microliters of anti-Akt antibody beads (Cell Signaling Technology, Danvers, MA, USA) was added to 200 μl of the cell lysate and incubated overnight at 4 °C with gentle rocking. The immunoprecipitates were washed twice with 500 μl of 1× cell lysis buffer and twice with 500 μl of 1× kinase buffer. The immunoprecipitates were resuspended in 50 μl of 1× kinase buffer mixed with 200 μM ATP and 1.5 μl of GSK-3 fusion protein and then incubated at 30 °C for 30 min. The mixtures were subjected to western blot analysis with phospho-GSK3β antibody (1:1000, Cell Signaling Technology, Danvers, MA, USA).

### Construction of plasmids

The *A3G* phosphorylation-site mutant plasmids (T32A, T32D, T32E, T218A, T218D, T218E, T32AT218A, T32AT218D, T32DT218A, and T32DT218D) were generated by site-directed mutagenesis from the pEGFP-C1-*A3G* plasmid using special primers (Supplementary Table S3). The *PTEN* CD mutant plasmids (CD1, CD1CD2, CD3) were cloned from the *PTEN* cDNA clone (OriGene, Rockville, MD, USA) using the following primers: CD1 top: 5′-TGCAGAATTCACATGACAGCCATCATCAAAGAG-3′, bottom: 5′-AGTACTCGAGCT TACCTTTAGCTGGCAGACCAC-3′; CD1CD2 top: 5′-TGCAGAATTCACATGACAG CCATCATCAAAGAG-3′, bottom: 5′-AGCACTCGAGCTTACTCCTCTACTGTTTTTGTG -3′; CD3 top: 5′-TGCAGAATTCGGATGGTAGAGGAGCCGTCAAAT-3′, bottom: 5′-AGCACTCGAGCTCAGACTTTTGTAATTTGTGTATGCTG-3′. The PCR products were inserted into the pCMV-Myc vector at Xho I / EcoR I restriction sites.

### Plasmid transfection

293T cells were seeded in 60 mm plates and transfected with the *A3G* mutant plasmids using Lipofectamine 2000 reagent (Invitrogen, Carlsbad, CA, USA) according to the manufacturer’s instructions. Eight micrograms of plasmid was added into a final volume of 500 μl of serum-free DMEM (Gibco, Gaithersburg, MD, USA); 20 μl of Lipofectamine 2000 was added into a final volume of 500 μl serum-free DMEM, incubated for 5 min and added to the former plasmid mixture. The mixture was incubated at room temperature for 20 min and added to the cells. The cells were incubated at 37 °C in a 5% CO_2_ atmosphere for 48 h.

### Statistical analysis

All data are presented as the mean ± SEM of triplicate experiments and were analyzed with GraphPad Prism 5 (GraphPad Software, La Jolla, CA). The significance of differences between the groups was assessed by Student’s *t*-test, a one-way ANOVA, or a χ^2^ test. The results were considered statistically significant if *P *< 0.05.

## Additional Information

**How to cite this article**: Wu, J. *et al.* The virus-induced protein APOBEC3G inhibits anoikis by activation of Akt kinase in pancreatic cancer cells. *Sci. Rep.* **5**, 12230; doi: 10.1038/srep12230 (2015).

## Supplementary Material

Supplementary Information
