# Supplementary material for: The virus-induced protein APOBEC3G inhibits anoikis by activation of Akt kinase in pancreatic cancer cells
Source: Sci Rep. 2015 Jul 16;5:12230. doi: 10.1038/srep12230 (PMC4503957; doi:10.1038/srep12230)
Supplement: Supplementary Information [file srep12230-s1.pdf]

The virus-induced protein APOBEC3G inhibits anoikis by activation of Akt kinase in pancreatic cancer cells

Jia Wu[1], Tian-Hui Pan[1], Song Xu [1], Li-Tao Jia[1,3], Lin-Lin Zhu[1], Jian-Shan Mao[1], Yong-Liang Zhu[1,2] & Jian-Ting Cai[1]

[1] Laboratory of Gastroenterology, Second Affiliated Hospital of Zhejiang University, School of Medicine, Hangzhou, China; [2] Cancer Institute and Education Ministry Key Laboratory of Cancer Prevention and Intervention, Zhejiang University School of Medicine, Hangzhou, China; [3] Department of Gastroenterology, Tongde Hospital of Zhejiang Province, Hangzhou, China.

Corresponding author: Dr. Yong-Liang Zhu, Laboratory of Gastroenterology, Second Affiliated Hospital of Zhejiang University School of Medicine, Hangzhou, Zhejiang Province, 310009, China. Tel: 86-571-87767061, Fax: 86-571-87214404, Email: drylzhu@163.com, or Dr. Jian-Ting Cai, Email: jtcai@live.cn.

Supplementary Table S1 Differently expressed genes in the global cDNA microarray

Gene Symbol	Ratio	Gene Symbol	Ratio	Gene Symbol	Ratio
ANKRD22	20.528	TMCC1	3.156	CCDC69	2.524
KRT20	9.074	SDR16C5	3.098	TNFRSF10D	2.524
CCL26	8.972	CXorf50B	3.098	SEPP1	2.523
TXNIP	8.891	CCDC69	3.095	TNFSF9	2.514
G0S2	8.336	EREG	3.063	PDZK1	2.507
CSF2	8.315	ANXA6	3.061	CDH17	2.505
APOBEC3G	6.756	ATP8A1	3.059	RAB26	2.501
EGR2	6.693	CARD16	3.023	JUNB	2.497
ARRDC4	6.185	LGALS7 /// LGALS7B	3.02	ABLIM3	2.493
PCK1	6.039	PLAC1	3.016	MLF1	2.488
LYZ	5.533	TMEM178	3.011	C7orf68	2.487
IL8	5.444	PTGR1	3.006	LYZ	2.487
EGR3	5.148	NMI	2.995	BCHE	2.483
MLF1	5.139	RGS2	2.99	SECTM1	2.479
ADM	5.069	LRCH3	2.989	VWDE	2.47
IGFBP3	4.937	SCN3A	2.981	FXYD2	2.458
CCL20	4.881	C3	2.961	PRSS1 /// TRY6	2.456
SERPINI1	4.776	SOD2	2.957	CD58	2.456
LPXN	4.67	SYCP2	2.937	HBA1 /// HBA2	2.455
LY96	4.663	ZFP36	2.928	PROCR	2.454
FOS	4.627	GMFG	2.91	OTUB2	2.451
UBE2L6	4.503	TBC1D8B	2.904	APOBEC3F	2.446
PSMB9	4.464	BHLHE41	2.874	LIF	2.428
FXYD2	4.403	GEM	2.859	ISG20	2.411
TBC1D4	4.374	TIGD7	2.853	ABAT	2.408
GBP1	4.248	HLA-DQB1	2.852	NOD2	2.408
TRIM31	4.247	SRPX	2.846	CTSH	2.407
SERPINA1	4.243	CD163L1	2.838	C17orf60	2.399
CCDC153	4.229	TIMP3	2.797	FBXO2	2.399
SLC5A3	4.213	EGR1	2.786	LOC728730	2.384
SGK1	4.131	HLA-DQB1	2.766	SLC5A3	2.38
HCP5	4.107	CXCL6	2.762	ZFAND2A	2.38
SERPINA1	4.088	GBP2	2.757	AREGB	2.374
KIAA1128	4.058	C10orf116	2.752	GRTP1	2.363
LEMD1	4.048	PRRG4	2.751	HOXA13	2.359
EGR4	3.962	SNAI2	2.748	MTRF1L	2.358
SERPINE2	3.859	WFDC3	2.74	DKK3	2.355
TFF2	3.833	TFPI	2.727	STXBP6	2.353
PRAP1	3.771	ECM1	2.714	C1orf161	2.353
FABP1	3.62	CTSS	2.709	DIRAS2	2.35
MAGEB6	3.608	FAM13A	2.704	CD44	2.342
CXCL1	3.599	CARD16 /// CASP1	2.7	LY75	2.339
CXCL2	3.579	TUBB2B	2.697	PTPRM	2.339
PPP4R4	3.561	DUSP1	2.691	DDIT3 /// NR1H3	2.337
GBP3	3.52	PER3	2.677	FGD5	2.337
TMEM154	3.487	SYTL4	2.676	JUN	2.334
ADSSL1	3.485	CCND2	2.672	BDH2	2.333
AREG	3.472	DKK3	2.666	MAGEB2	2.329
GABBR1 /// UBD	3.441	SLC20A1	2.665	SAT1	2.327
HSPA6	3.402	EAF2	2.661	JUN	2.322
LRRC70	3.401	TRIM34 /// TRIM6-TRIM34	2.657	CD58	2.321
ANKRD37	3.392	RASGEF1A	2.651	PSMB8	2.321
GDF15	3.374	JUN	2.643	ISG20	2.317
GIMAP2	3.372	CDKN1A	2.635	ZNF226	2.316
FHL1	3.369	TCAM1	2.635	BHLHE41	2.315
DHRS2	3.343	TMEM45B	2.634	ATP2B4	2.313
EGR1	3.337	ITPR1	2.628	NFKBIZ	2.311
KL	3.332	TNNT1	2.603	CST6	2.305
DDIT4	3.315	EGR1	2.597	NRP2	2.304
CREBZF	3.31	NAGS	2.592	EFNA4	2.302
ALPK1	3.259	CYP3A5	2.585	OAS1	2.3
EPAS1	3.248	HECA	2.581	TMEM45B	2.299
EHF	3.243	GCOM1	2.575	MXD1	2.288
DUSP2	3.236	BEAN	2.573	SLC22A4	2.278
CSPG5	3.231	PRSS2	2.565	AK7	2.277
ATP6V0A4	3.228	HCN1	2.555	ABAT	2.268
SRGN	3.189	PRSS1	2.536	IGF2 /// INS-IGF2	2.26

Gene Symbol	Ratio
C10orf125	2.256
PSD2	2.253
ZFP14	2.234
CAMK2N1	2.233
JAKMIP3	2.228
BCL3	2.226
TNFAIP3	2.224
FAM113A	2.222
TGM2	2.22
WSB1	2.215
HEMGN	2.215
GDF15	2.213
JUN	2.211
PRSS3	2.206
KLF11	2.202
SPAG4	2.201
TFPI	2.2
PEX13	2.2
FAM13A	2.195
BTG2	2.184
COL6A1	2.181
PAQR5	2.181
ASPHD2	2.169
WWTR1	2.164
C1orf58	2.164
DOCK8	2.162
CDC2L6	2.162
E2F3	2.161
TFPI	2.147
DAZAP2	2.145
DUSP5	2.136
SMOC2	2.133
RNF183	2.127
SLC5A3	2.126
VWA5A	2.125
TIMP3	2.121
GIMAP6	2.121
CSRNP1	2.119
TRPC1	2.118
TFPI	2.115
SORBS1	2.111
TAF5	2.109
SFRS11	2.108
CAB39L	2.106
CAMK2N1	2.103
S100A2	2.101
HGD	2.1
TSPAN8	2.099
AP1S2	2.098
FAM114A1	2.097
BOLA1	2.097
DNAJC12	2.096
DKK3	2.093
OTUB2	2.087
CEACAM1	2.077
AKR1C1	2.073
RRAS	2.072
WDR5B	2.072
NAV2	2.071
IFIH1	2.066
RAB26	2.065
PLAU	2.064
SLC6A1	2.062
PDK4	2.061
CALB2	2.055
PMS2L1	2.052
STXBP6	2.049

Gene Symbol	Ratio
PKIB	2.045
APOC2 /// APOC4	2.044
PPIC	2.041
BIRC3	2.041
SPINK7	2.039
ZNF818P	2.034
CD58	2.033
IL8	2.032
EPAG	2.029
FGFR3	2.023
CXCL3	2.022
SLC2A1	2.018
GPR160	2.011
ARRB1	2.01
ERAP1	2.008
CHD2	2.007
PTK6	2.005
HLA-DQB1	2.
FOXP1	0.499
SOX2OT	0.498
HIST1H1B	0.496
PPL	0.495
ASNS	0.495
SPATA1	0.495
WNT11	0.491
JUB	0.491
HIST3H2A	0.49
IGF1R	0.488
TMPO	0.488
GALNT7	0.486
SFRS2B	0.484
MATR3	0.482
HMGB3	0.48
PHF17	0.48
CYFIP2	0.479
SFRS2B	0.479
TMEM133	0.47
COL2A1	0.463
RPS20	0.461
ALDH1A2	0.459
ILF3	0.457
ELOVL6	0.453
HIST1H2AC	0.452
ADAL	0.452
NCRNA00153	0.45
ZNF124	0.449
KIAA1659	0.449
SOX2	0.449
GPR177	0.448
KCNE3	0.446
CHM	0.443
FRAS1	0.439
DENND1A	0.438
RPL27A	0.437
KRTAP3-3	0.437
SYCP3	0.433
ROR1	0.43
ELOVL6	0.426
MX2	0.423
RPL13	0.422
MTMR1	0.418
FILIP1	0.417
NUP62	0.41
KCNQ5	0.409
IL18	0.407
SHANK2	0.402
RIN2	0.39

Gene Symbol	Ratio
POF1B	0.387
ITPR2	0.383
CNNM2	0.372
SLC25A31	0.37
HIST1H1E	0.33
PCDH9	0.33
ITPR2	0.321
FAM43A	0.319
AMOTL2	0.318
THBS1	0.315
SDPR	0.302
SEMA6A	0.29
MIRHG1	0.273
LPCAT1	0.26
SEMA6A	0.26
ABCA13	0.155
SDPR	0.117

Supplementary Table S2 Basic information on the pancreatic cancer patients (n=54)

Variable	Data
Age,y	
Mean(SD)	61.3 (8.2)
Range	36-77
Sex	
Male	30
Female	24
CA199,U/mL	
<=37	9
>37	41
Tumor size, cm	
<=2	6
>2	45
Location	
Head of the pancreas	39
Body and tail of pancreas	15
TNM stage	
I	15
II	10
III	27
IV	2
Differentiation grade	
Poorly deferentiation	14
Moderately differentiation	37
Well differentiation	22
Pathological type	
Adenocarcinoma	49
Adenosquamous carcinoma	2
Mucinous cystadenocarcinoma	3

Supplementary Table S3 Primers for site-directed mutagenesis of *A3G*

Sites	Primers
T32A	top 5’-ATCCTTTCTCGTCGGAATGCCGTCTGGCTGTGCTACGAA-3’<br>bottom 5’-TTCGTAGCACAGCCAGACGGCATTCCGACGAGAAAGGAT-3’
T32D	top 5’-ATCCTTTCTCGTCGGAATGACGTCTGGCTGTGCTACGAA-3’<br>bottom 5’-TTCGTAGCACAGCCAGACGTCATTCCGACGAGAAAGGAT-3’
T32E	top 5’-ATCCTTTCTCGTCGGAATGAAGTCTGGCTGTGCTACGAA-3’<br>bottom 5’-TTCGTAGCACAGCCAGACTTCATTCCGACGAGAAAGGAT-3’
T218A	top 5’-GTCAGAGGACGGCATGAGGCTTACCTGTGTTATGAGGTG-3’<br>bottom 5’-CACCTCATAACACAGGTAAGCCTCATGCCGTCCTCTGAC-3’
T218D	top 5’-GTCAGAGGACGGCATGAGGATTACCTGTGTTATGAGGTG-3’<br>bottom 5’-CACCTCATAACACAGGTAATCCTCATGCCGTCCTCTGAC-3’
T218E	top 5’-GTCAGAGGACGGCATGAGGAATACCTGTGTTATGAGGTG-3’<br>bottom 5’-CACCTCATAACACAGGTATTCCTCATGCCGTCCTCTGAC-3’

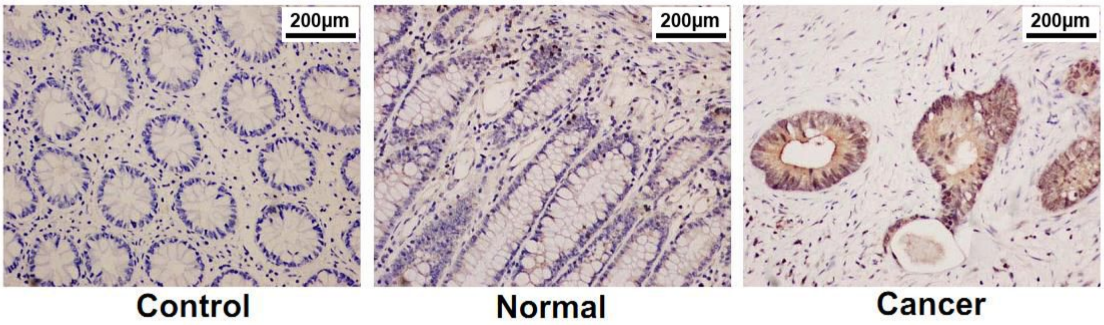

Supplementary Figure S1 Immunohistochemical staining
Slides from 23 matched human colon cancer and para-cancerous tissues were detected by immunohistochemical staining.

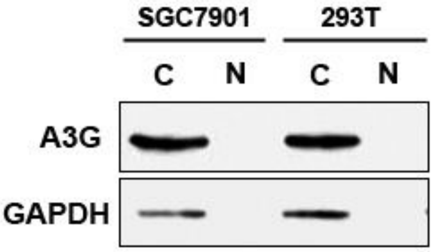

Supplementary Figure S2 A3G distributed in cytoplasm
A3G was transfected into SGC7901 and 293T cells respectively, nucleus and cytoplasm were separated and subsequently detected by western blot (C: Cytoplasm; N: Nucleus). GAPDH was used as a loading control.

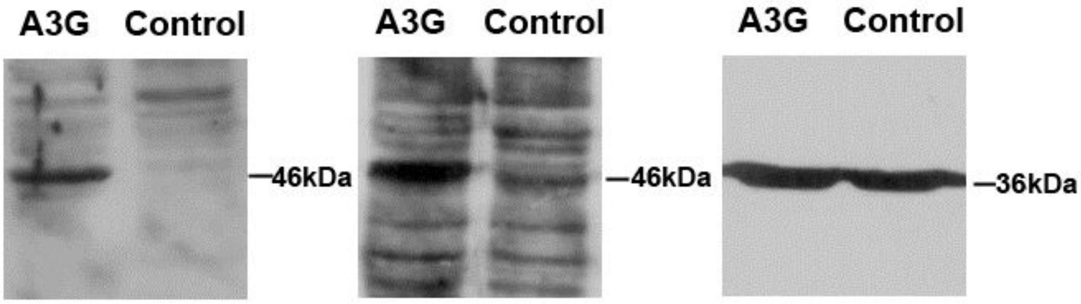

Supplementary Figure S3 Full length western immunoblots as presented in Figure 2C. The left immunoblot represents HA-tag staining. The middle immunoblot represents A3G staining. The right immunoblot represents GAPDH staining.

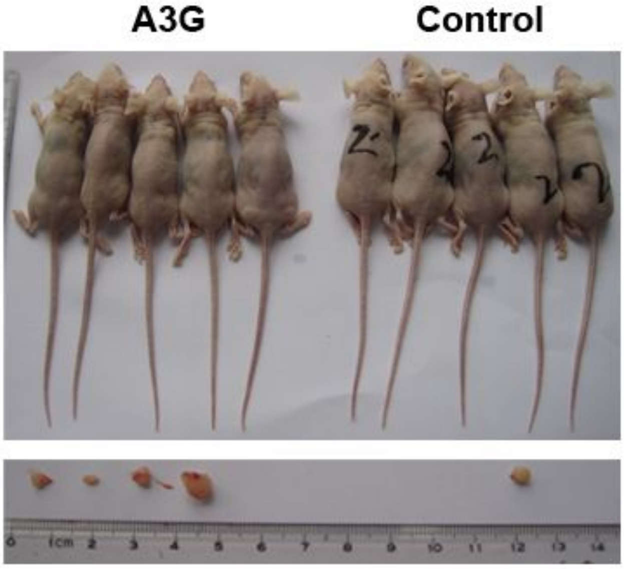

Supplementary Figure S4 Compared with the control, 1×10^5 quantity of the A3G-expressing group could form more xenograft tumor in nude mice after 4 weeks. Two groups' photograph of tumors is shown.

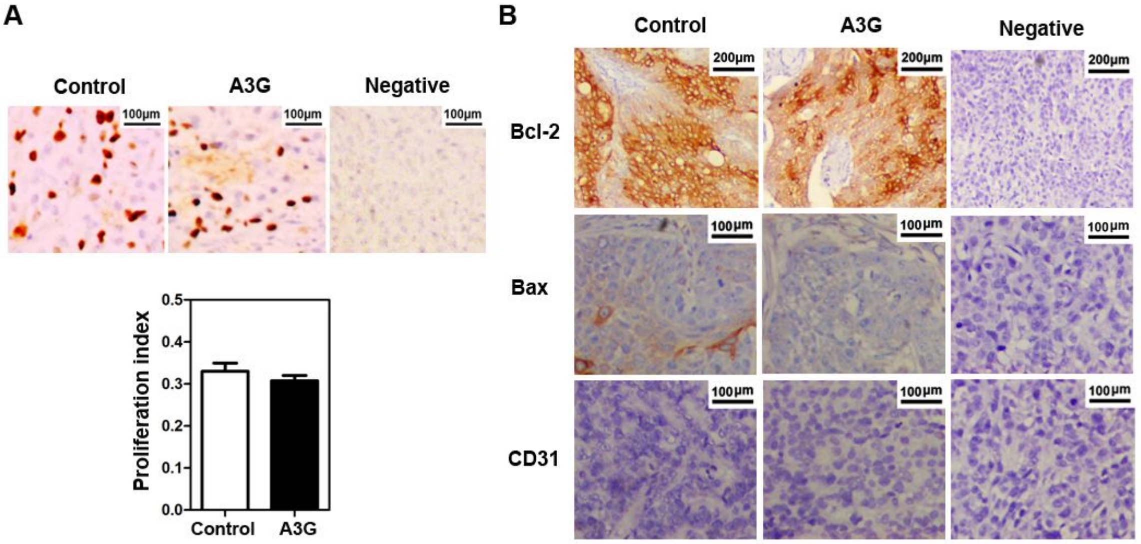

Supplementary Figure S5 Immunostaining analysis of Ki67, Bcl-2, Bax and CD31 of xenograft tumors respectively generated by A3G-expressing cells and control cells in nude mice for 4 weeks. **A**. Percentage of Ki67-positive cells. P >0.05, χ^2 test; **B**. Detection of Bcl-2, Bax and CD31 in A3G-expressing group.

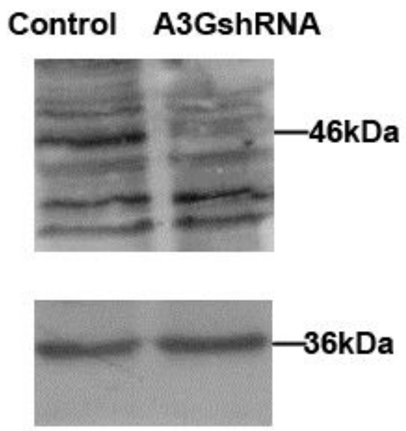

Supplementary Figure S6 Full length western immunoblots as presented in Figure 3B. The top immunoblot represents A3G staining. The bottom immunoblots represent GAPDH staining.

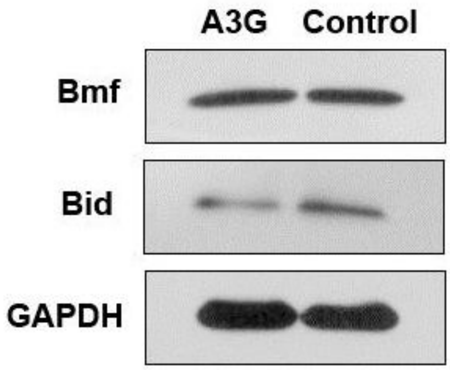

Supplementary Figure S7 Detection of Bmf and Bid. Western blot showed that Bid was down-regulated in stable A3G expressing cells, and Bmf was not changed. All data are repeated for three times. GAPDH was used as a loading control.

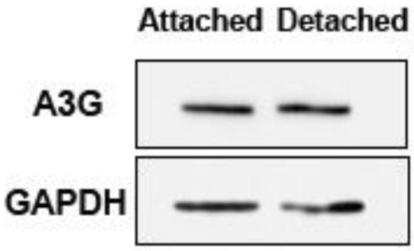

Supplementary Figure S8 Detection of A3G in attached and detached cells. GAPDH was used as a loading control.

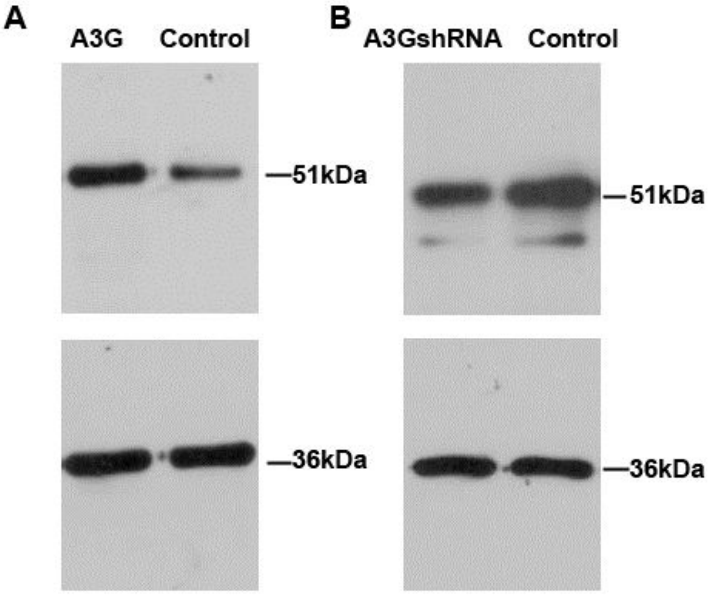

Supplementary Figure S9 Full length western immunoblots as presented in Figure 4B. The A top represents phospho-GSK3β staining and the A bottom represents GAPDH staining for overexpression of A3G. The B top represents phospho-GSK3β staining and the B bottom represents GAPDH staining for knockdown of A3G.

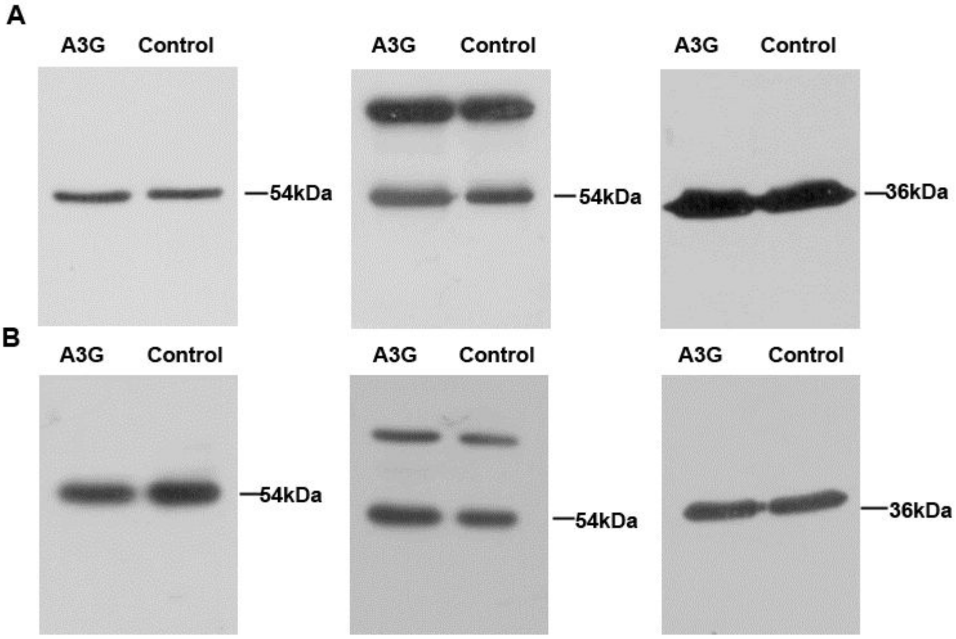

Supplementary Figure S10 Full length western immunoblots as presented in Figure 4E. The A left represents PTEN staining, A middle represents phosphor-PTEN staining and A right represents GAPDH staining in BxPC3 cells. The B left represents PTEN staining, B middle represents phosphor-PTEN staining and B right represents GAPDH staining in SGC7901 cells.

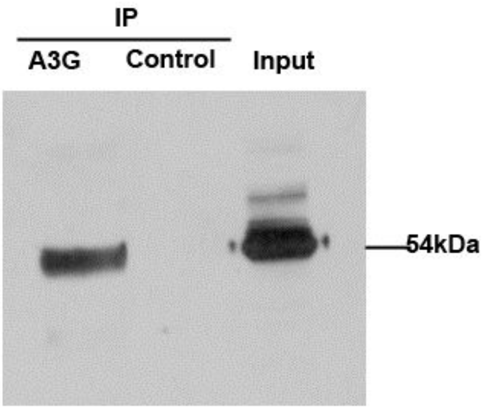

Supplementary Figure S11 Full length western immunoblots as presented in Figure 4F. The immunoblot represents PTEN staining.

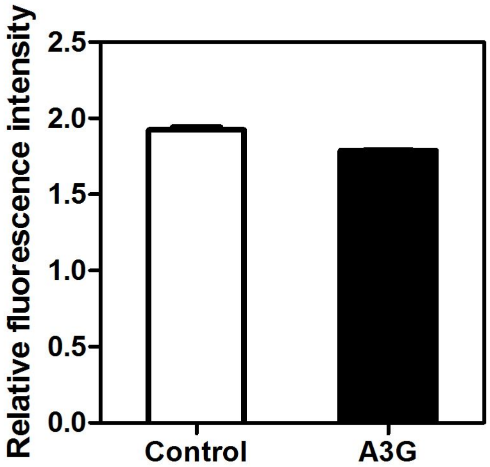

Supplementary Figure S12 Up-regulation of A3G not affects the NF-κB activity. *A3G* was co-transfected into 293T cells with the NF-κB/pGL2 luciferase reporter plasmid, and luciferase activity was assayed 24 h later (*P<0.05, **P<0.01; Student's *t*-test). All data are represented as the means ± SEM of triplicate experiments.
